# Cerebral salt wasting syndrome in an elderly patient with traumatic brain injury: a case report

**DOI:** 10.3389/fmed.2026.1809469

**Published:** 2026-04-14

**Authors:** Pengpeng Li, Yangyang Gao, Junfeng Li, Shaohua Lin, Zehong Zhang, Lei Luo, Wei Liu

**Affiliations:** 1 Department of Neurosurgery, Xi’an Aerospace Hospital of Northwest University, Xi’an, Shanxi, China; 2 Department of Neurosurgery, Ningxia Medical University, Yinchuan, China

**Keywords:** altered mental status, Cerebral Salt Wasting Syndrome, coma, hyponatremia, traumatic brain injury

## Abstract

**Background:**

Cerebral Salt Wasting Syndrome (CSWS) represents a diagnostically challenging and potentially fatal complication of traumatic brain injury (TBI). It is defined by renal sodium excretion-induced hyponatremia accompanied by hypovolemia, yet its clinical signature—worsening consciousness—frequently mimics intracranial hypertension or Syndrome of Inappropriate Antidiuretic Hormone Secretion (SIADH). Misdiagnosis, particularly the imposition of fluid restriction, may precipitate catastrophic deterioration.

**Case presentation:**

An 83-year-old male with pre-existing hypertension and atrial fibrillation presented with moderate-to-severe TBI (GCS 8) following a fall, with imaging revealing cerebral contusions, subarachnoid hemorrhage, and subdural hematoma. Initial conservative management yielded progressive neurologic recovery (GCS 13 by day 7). On day 8, however, he abruptly lapsed into coma. Urgent cranial CT demonstrated interval resolution of hemorrhagic lesions without new mass effect or hydrocephalus. Biochemical analysis uncovered severe hyponatremia (123.5 mmol/L). CSWS was diagnosed based on clinical trajectory, volume status assessment, laboratory parameters, and exclusion of SIADH. Prompt intervention with hypertonic saline and intravascular volume repletion produced rapid neurologic recovery.

**Conclusion:**

This case illuminates CSWS as an insidious mimic of primary neurologic deterioration in TBI patients, particularly the elderly with cardiovascular comorbidities. Hyponatremia must be investigated as a reversible cause of unexplained coma in this population. Accurate differentiation from SIADH is critical to avert iatrogenic harm, and timely, monitored sodium correction can achieve dramatic reversal of life-threatening neurologic deficits.

## Introduction

Traumatic brain injury (TBI) constitutes a major global health burden, with elderly patients facing disproportionately higher risks of morbidity and mortality due to pre-existing comorbidities and diminished physiological reserve (1). The management of secondary complications is as crucial as addressing the primary injury. Hyponatremia is a common electrolyte disturbance following TBI, with Syndrome of Inappropriate Antidiuretic Hormone Secretion (SIADH) and Cerebral Salt Wasting Syndrome (CSWS) being the principal etiologies (2).

Despite pathophysiological distinctions, CSWS is often underrecognized or misdiagnosed as SIADH. CSWS is characterized by renal sodium and water loss leading to hypovolemic hyponatremia, likely mediated by increased secretion of natriuretic peptides (3). In contrast, SIADH involves euvolemic or hypervolemic hyponatremia due to inappropriate water retention. This distinction is therapeutic; CSWS requires sodium and volume replacement, while SIADH is managed with fluid restriction (4). Misdiagnosis can lead to inappropriate treatment, exacerbating cerebral hypoperfusion and ischemia.

We present a case of CSWS in an elderly male with severe TBI, highlighting the diagnostic challenge and the dramatic clinical response to targeted therapy. This report emphasizes the importance of prompt recognition and management to prevent neurological deterioration.

## Case presentation

An 83-year-old Han Chinese male with a history of hypertension, coronary artery disease, atrial fibrillation, and unspecified hyperglycemia was transferred to our emergency department after a ground-level fall. He sustained a rear occipital impact with immediate loss of consciousness and multiple episodes of vomiting.

### Initial examination

On admission, vital signs were stable. Neurological examination revealed a GCS score of 8 (E1V2M5). Pupils were equal and reactive to light. Bilateral Babinski signs were equivocal. Initial laboratory tests were significant for elevated random blood glucose (13.15 mmol/L) and elevated D-dimer.

### Imaging findings

**Figure 1:**
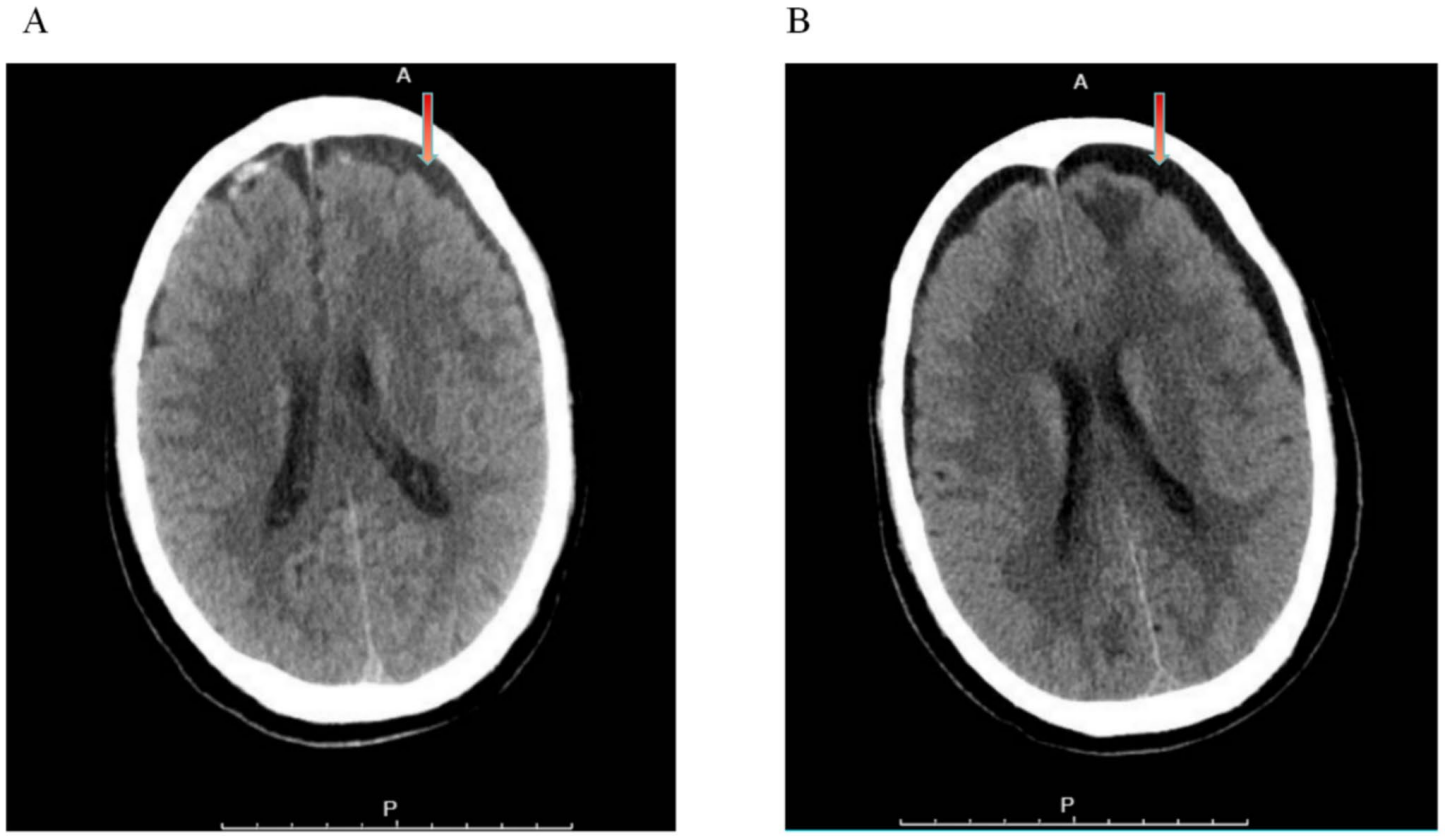
Serial non-contrast computed tomography (NCCT) of the brain. **(A)** Admission NCCT scans. Axial images demonstrate bilateral frontal hypodense fluid collections with hyperdense sediment levels (red arrows). These findings are consistent with pre-existing chronic subdural hygromas with acute traumatic hemorrhage, a common phenomenon in the elderly. Acute cerebral contusions are also visible in the bilateral frontal and temporal regions. **(B)** Follow-up NCCT on hospital day 8 (during neurological decline). Axial image at a comparable level shows stable post-traumatic changes with resolving hemorrhage. There is no evidence of new mass effect, midline shift, or herniation to explain the acute deterioration in consciousness.

Non-contrast computed tomography (NCCT) of the brain revealed bilateral frontal and temporal contusions, traumatic subarachnoid hemorrhage, bilateral frontal subdural hematomas with fluid levels, basilar skull fracture, and occipital bone fracture (Figure 1A). Chest CT suggested pulmonary congestion and cardiomegaly.

### Hospital course

The patient received edaravone as a neuroprotective agent for 7 days. Mannitol was administered intravenously every 8 h for the first 3 days to control intracranial pressure. Levetiracetam was given as prophylactic antiepileptic therapy for 3 days. His neurological status gradually improved, reaching a GCS of 13 (E4V4M5) by day 7. Enteral nutrition was initiated via nasogastric tube.

### Development of CSWS

On hospital day 8, the patient experienced a sudden decline in consciousness. Clinically, he presented with tachycardia (heart rate 103–112 bpm) in the setting of known atrial fibrillation. Repeat NCCT brain showed expected resolution of hemorrhagic lesions without mass effect or new injury (Figure 1B). However, serum sodium plummeted to 123.5 mmol/L (from 138 mmol/L on admission). Other laboratory findings included serum osmolarity 255 mOsm/kg, urine osmolarity 610 mOsm/kg, spot urine sodium >80 mmol/L, elevated UR acid 6.8 mg/dL, and elevated BUN/creatinine ratio. Central venous pressure measurement was 3 cm H₂O (low-normal). These findings were consistent with hypovolemic hyponatremia, supporting the diagnosis of CSWS.

### Treatment and outcome

Management involved intravenous administration of 3% hypertonic saline (150 mL over 4 h) followed by continuous infusion, coupled with aggressive oral and intravenous sodium chloride supplementation. Fluid intake was liberalized to maintain euvolemia. Serum sodium was monitored every 4–6 h. The patient’s neurological status improved dramatically within 24 h as sodium levels corrected to 135 mmol/L. He was subsequently weaned off hypertonic saline and maintained on oral salts. No central pontine myelinolysis occurred. His hospital course was later complicated by healthcare-associated pneumonia, which was treated with antibiotics (see Table 1).

**Table 1:** Sequential laboratory parameters during CSWS diagnosis and management.

Parameter	Admission (day 1)	Day 7 (pre-decline)	Day 8 (diagnosis)	Day 9 (post-treatment)	Reference range
Serum sodium (mmol/L)	138	136	123.5	135	137–145
Serum osmolality (mOsm/kg)	—	—	255	285	275–295
Urine sodium (mmol/L)	—	—	>80	45	<20
Urine osmolality (mOsm/kg)	—	—	610	380	50–1200
BUN (mg/dL)	12.5	11.8	18.2	14.1	7–20
Serum uric acid (mg/dL)	5.1	4.9	6.8	5.5	3.5–7.2

## Discussion

This case exemplifies a classic yet challenging presentation of CSWS masquerading as neurological deterioration in an elderly patient with TBI. The rapid resolution of symptoms with sodium and volume repletion provides compelling evidence for the diagnosis and underscores the perils of misdiagnosis.

Several case reports have documented CSWS in older adults mimicking stroke or TBI progression, leading to diagnostic delays (5, 6). The pathophysiological mechanism of CSWS remains incompletely elucidated but is strongly linked to dysregulation of natriuretic peptides, such as atrial natriuretic peptide (ANP) and brain natriuretic peptide (BNP), following brain injury (7). These peptides promote renal sodium and water excretion, leading to volume depletion, which in turn stimulates ADH release as a secondary phenomenon. This creates a complex picture with biochemical features overlapping with SIADH (e.g., high urine osmolality and high urine sodium) but with a diametrically opposed volume status (8). Key differentiating factors in our patient were the presence of hypovolemia (inferred from high BUN/creatinine ratio and clinical context) and the dramatic response to volume expansion. The elevated serum uric acid further supported CSWS over SIADH, where it is typically low (9). Fractional excretion of urate (FEurate) was not measured due to hospital limitations.

A practical diagnostic approach to differentiate CSWS from SIADH includes three key elements. First, volume status assessment shows hypovolemia in patients with CSWS, while those with SIADH present with euvolemia or hypervolemia. Second, laboratory findings in CSWS typically reveal an elevated BUN to creatinine ratio and normal or elevated serum uric acid levels, whereas SIADH is associated with low uric acid levels. Third, response to fluid challenge differs markedly, with volume expansion leading to clinical improvement in CSWS but providing no benefit or even causing worsening in SIADH. Bedside tools such as central venous pressure monitoring and daily weight measurements are useful for dynamic volume assessment.

Management of CSWS hinges on aggressive sodium and fluid replacement. Hypertonic saline is the cornerstone for rapid correction in symptomatic patients (10). However, in elderly patients with cardiac history, this poses a significant challenge due to the risk of precipitating heart failure. Our approach involved meticulous monitoring of sodium levels (every 4–6 h), central venous pressure, and clinical status to balance the need for rapid correction against the risk of volume overload. Fludrocortisone, a mineralocorticoid, is a useful adjunctive therapy to promote renal sodium reabsorption and reduce the required fluid volume (11), though it was not used in this case due to concerns about hyperglycemia.

This case highlights several learning points: (1) CSWS can occur even in cases of stable or improving radiographic findings, (2) serial sodium monitoring is imperative in the first 2 weeks after TBI, especially in elderly patients, and (3) the treatment for CSWS (volume expansion) is the opposite of that for SIADH (fluid restriction), making accurate diagnosis critical to avoid iatrogenic harm.

## Conclusion

CSWS is a potentially catastrophic but treatable cause of neurological decline following TBI. Clinicians must maintain a high index of suspicion for CSWS in any brain-injured patient with hyponatremia and worsening mental status. Rapid diagnosis based on clinical assessment and targeted laboratory tests, followed by prompt and carefully monitored administration of sodium and fluids, is essential to reverse neurological deficits and improve outcomes. This case serves as a crucial reminder of the importance of electrolyte management in neurocritical care.

## Data Availability

The original contributions presented in the study are included in the article/supplementary material, further inquiries can be directed to the corresponding author.
